# Identifying Cancer Subtypes Using a Residual Graph Convolution Model on a Sample Similarity Network

**DOI:** 10.3390/genes13010065

**Published:** 2021-12-27

**Authors:** Wei Dai, Wenhao Yue, Wei Peng, Xiaodong Fu, Li Liu, Lijun Liu

**Affiliations:** 1 Faculty of Information Engineering and Automation, Kunming University of Science and Technology, Kunming 650050, China; dw@cnlab.net (W.D.); yuewenhao_12@163.com (W.Y.); xiaodong_fu@hotmail.com (X.F.); ieall@kmust.edu.cn (L.L.); cloneiq@126.com (L.L.); 2 Computer Technology Application Key Lab of Yunnan Province, Kunming University of Science and Technology, Kunming 650050, China

**Keywords:** residual graph convolutional network, cancer subtype classification, deep learning, sample interaction

## Abstract

Cancer subtype classification helps us to understand the pathogenesis of cancer and develop new cancer drugs, treatment from which patients would benefit most. Most previous studies detect cancer subtypes by extracting features from individual samples, ignoring their associations with others. We believe that the interactions of cancer samples can help identify cancer subtypes. This work proposes a cancer subtype classification method based on a residual graph convolutional network and a sample similarity network. First, we constructed a sample similarity network regarding cancer gene co-expression patterns. Then, the gene expression profiles of cancer samples as initial features and the sample similarity network were passed into a two-layer graph convolutional network (GCN) model. We introduced the initial features to the GCN model to avoid over-smoothing during the training process. Finally, the classification of cancer subtypes was obtained through a softmax activation function. Our model was applied to breast invasive carcinoma (BRCA), glioblastoma multiforme (GBM) and lung cancer (LUNG) datasets. The accuracy values of our model reached 82.58%, 85.13% and 79.18% for BRCA, GBM and LUNG, respectively, which outperformed the existing methods. The survival analysis of our results proves the significant clinical features of the cancer subtypes identified by our model. Moreover, we can leverage our model to detect the essential genes enriched in gene ontology (GO) terms and the biological pathways related to a cancer subtype.

## 1. Introduction

Cancer is a heterogeneous disease initiated by random somatic mutations and driven by multiple genomic alterations [1,2,3]. Cancer patients are usually divided into subtypes according to their molecular profiles for effective cancer treatment [4,5,6]. Different subtypes of cancer patients have different clinical phenotypes, tumor morphologies, therapeutic schedules and prognoses [7]. For example, breast cancer is divided into four subtypes: Luminal A, Luminal B, Basal and HER2. Each subtype has different forms and different reactions to drugs [8,9,10,11]. Accurate cancer subtyping can help understand the pathogenesis of cancer, boost clinical treatment, improve patients’ survival rate and advance research on cancer genomics and precision medicine [12]. In the past decade, some large-scale cancer genomics projects have published genomic, epigenomic, transcriptomic, and proteomic data from thousands of cancer patients [13]. These projects include the cancer genome atlas (TCGA) [14], the International Cancer Genome Consortium (ICGC) [15], and the Pan-Cancer Analysis of Whole Genomes (PCAWG) [16]. These cancer genomic data have extensively promoted the development of computational methods for cancer subtype classification by integrating multi-omic data. Previous studies [17] showed that the gene expression data perform best in detecting the cancer subtypes compared with other omics data. Hence, considering the availability of the gene expression data for most patients, we focused on designing a novel computational method for cancer subtype classification based on gene expression data.

Recently, different computational methods have been proposed to detect cancer subtypes [18,19,20]. These methods usually build on feature engineering to cluster or classify patients into several groups. Due to the high dimension and small sample size of biological data, early approaches reduced the sample’s features to a reasonable extent and used those features to cluster cancer subtypes. iCluster [21] reduces the dimensionality of datasets to learn features while simultaneously incorporating the flexibility of the associations between different data types and the variance-covariance structure within data types. SparseK [22] leverages a lasso-type penalty to select features adaptively. Non-negative matrix factorization [23,24] methods usually decompose the sample feature matrix into two low-rank matrices, where one denotes the feature representation in a low dimension space, and the other indicates the potential clusters. One study [25] comprehensively reviewed recent methods for clustering cancer subtypes.

With the development of deep learning techniques, some deep learning methods, such as convolutional neural networks (CNNs) [26,27,28], generative adversarial models (GANs), deep autoencoders (DANs), restricted Boltzmann’s machine (RBM), convolutional autoencoders (CAE), stacked autoencoders (SAE) [29], variational autoencoders (VAE) [30], recurrent neural networks (RNNs), long short-term memory (LTSM), multi-scale convolutional neural networks (M-CNN), and multi-instance learning convolutional neural networks (MIL-CNN) have been applied to the cancer research field [31,32]. The classifying-based methods usually train a model on some cancer samples with known subtype labels and then employ the model to predict subtypes for new cancer samples. According to the feature extraction and classification differences, the classification-based methods fall into two categories, two-stage methods and end-to-end methods [33]. The two-stage processes take two separate steps to learn the sample features and to make predictions. Fakoor et al. [34] used PCA to address the very high dimensionality of the initial raw feature space, followed by sparse feature learning techniques (sparse autoencoders) to construct the discriminative and sparse features for the final classification step. Stacked Autoencoders (SAE) [29] and Variational Autoencoders (VAE) [30] have been applied to extract sample features and then to input these features into a classifier to obtain cancer subtypes. Subtype-GAN [17] is a deep adversarial learning approach that uses a neural network to model complex omics data accurately. With the latent variables extracted from the neural network, Subtype-GAN uses consensus clustering and the Gaussian Mixture model to identify cancer samples’ molecular subtypes. Those models can automatically capture the data structure and achieve good prediction performance. However, the major issue with two-stage methods is that there is no guarantee that the features retrieved at the first stage can support the cancer subtype classification. The end-to-end approaches learn and classify samples simultaneously. DeepType [35] inputs cancer sample data into a multi-layer neural network to reduce the data dimension. Meanwhile, it joins supervised classification with unsupervised clustering to determine the cancer-relevant data representation with the cluster structure.

The methods mentioned above only used the cancer sample data to input into models for feature learning, and they did not consider the relationship between cancer samples. We believe that the rich information between samples can promote the classification of cancer subtypes. The Graph Convolutional Network (GCN) model is an efficient method to mine valuable information from the network, which converts the nodes in the network to low dimension vectors while maximally preserving the original features and the local network structure. Recently, the GCN model has been wildly applied in bioinformatics, such as driver gene identification [13] and drug sensitivity prediction [36]. Lee et al. [37] implemented a graph convolution operation on multiple pathway-gene networks to learn gene features, and then leveraged a multi-attention based ensemble model to combine these features in several hundreds of pathways to identify cancer sample subtypes. In this work, we designed an end-to-end deep learning method, namely ERGCN, which uses a cancer sample similarity network and a Residual Graph Convolutional Network to classify cancer subtypes based on gene expression profiles. Our model utilizes the gene expression profiles as sample features and constructs a sample similarity network regarding their gene co-expression patterns. Then, the sample features and sample similarity network are put into a residual graph convolutional network model to learn the feature representation of samples and to predict subtypes in an end-to-end way. We applied our model to predict subtypes for breast, GBM and lung cancer samples. The results show that our method achieves relatively better performance than the state-of-the-art models in terms of both internal and external evaluation metrics. Moreover, by analyzing the prediction results for breast cancer, we found some key genes that may cause the occurrence of breast cancer subtypes, demonstrating the practicality of ERGCN.

## 2. Materials and Methods

**Figure 1:**
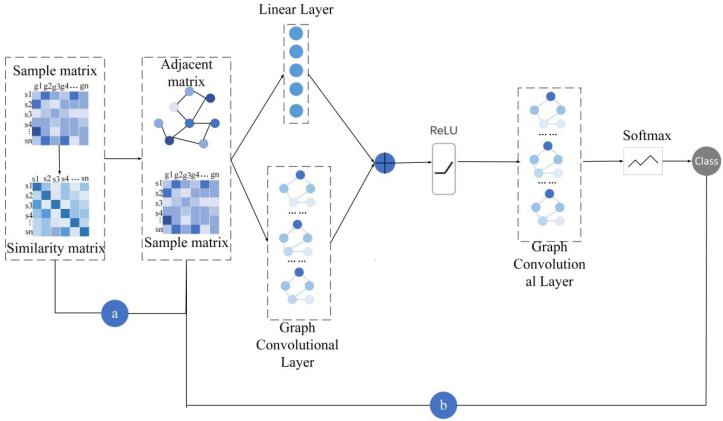
Framework of the ERGCN. (**a**) Calculate sample similarity matrix according to the Pearson correlation coefficient of their gene expression data and construct the sample adjacency matrix. (**b**) Input the sample features and the sample adjacency matrix into the residual graph convolution model to obtain category prediction.

Broadly, ERGCN takes two steps to identify cancer subtypes of cancer patients based on their gene expression data and the residual graph convolutional network. Firstly, it calculates the similarity between cancer patients and constructs a network where nodes are cancer patients and edges connect two nodes if their similarities are above a predefined threshold. Next, a residual graph convolutional neural network algorithm is adopted to diffuse the node feature information through the network and to learn the feature representation for every node in an end-to-end pattern. At this step, ERGCN predicts the cancer subtypes of patients according to their feature representations. Figure 1 shows an overview of ERGCN for identifying cancer subtypes.

### 2.1. Datasets

We tested our model on three different cancer types from TCGA. These three cancer types included breast invasive carcinoma (BRCA), with 102 samples, glioblastoma multiforme (GBM), with 213 samples, and lung cancer (LUNG), with 85 samples. We downloaded the gene expression data and survival information for the three cancer types in supplementary files of Wang [38]. The cancer subtype information was retrieved from TCGA using the R package TCGAbiolinks [39]. By matching the sample ID of the gene expression data, we joined the sample gene expression data with their cancer subtypes. Hence, there were four cancer subtypes for the 102 breast cancer patients (e.g., Basal, LumA, LumB, Her2), four cancer subtypes for the 213 GBM cancer patients (e.g., LGr1, LGr2, LGr3, LGr4) and four cancer subtypes for the 85 lung cancer patients(e.g., Basal, Classical, Secretory, Primitive).

### 2.2. Network Construction

We constructed a patient network based on the similarity of patients. The similarity of patients was calculated by the Pearson correlation coefficient (PCC) [40] of their gene expression profiles (see Equation (1)).

(1) $$r=\frac{\sum_{i}^{n}\;\left(X_{i}-\bar{X}\right)\left(Y_{i}-\bar{Y}\right)}{\sqrt{\sum_{i}^{n}\;{\left(X_{i}-\bar{X}\right)}^{2}}\sqrt{\sum_{i}^{n}{\left(Y_{i}-\bar{Y}\right)}^{2}}}$$

where n is the number of genes, X_i_ and Y_i_ are the expression values of gene i for patients X and Y and $\bar{X}$ and $\bar{Y}\;$ are the average gene expression values of X and Y. The Pearson correlation coefficient (r) measures the degree of linear correlation between two patients, ranging from −1 to 1. When the absolute value of r nears 1, two patients are positively or negatively correlated. Otherwise, the value nears 0. Hence, we consider that an edge connects two patients and thus set the corresponding values in the adjacency matrix to 1 if the absolute value of the Pearson coefficient between the two samples was greater than the threshold (θ). Otherwise, no edges connected two patients, and the corresponding adjacency matrix values were zeros. In this work, θ was set to 0.42 for BRCA, 0.41 for LUNG and 0.8 for GBM.

### 2.3. Residual Graph Convolutional Neural Network

After constructing the patient network, we adopted a graph convolutional network (GCN) to learn the feature representations of the network nodes by considering the network structure and node attributes. A GCN model requires two inputs, an adjacent matrix storing the node connections and a node initial attribute matrix. Let G = (V, E) be a patient network, where V (|V| = n) and E are sets of nodes and edges, respectively. The relationship between each node forms an n × n adjacency matrix A. We assume that every node connects to itself. Hence, the diagonal elements of A are set to 1. D is the degree matrix of A, X is an n × d initial attribute matrix of network nodes, and d is the length of the node’s attributes. We regard the patient’s gene expression data as the initial node attributes. The GCN model gathers features for a node from itself and local neighbors, then propagates information among nodes and the network. Mathematically, a single GCN layer is defined as follows.

(2) $$H^{\left(l+1\right)}=p\left(D^{-\frac{1}{2}}{\mathrm{AD}}^{-\frac{1}{2}}H^{\left(l\right)}W^{\left(l\right)}\right)$$

where H^(l+1)^ represents node features learned by the (l + 1)th GCN layer, $W\in R^{n*h}\;$ is a weight parameter matrix of the neural network, *h* is the hidden layer dimension, H^(l)^ comes from the output feature embedding of the previous GCN layer, H^(0)^ is initialized with X for the first GCN layer, and p is the nonlinear activation function.

To avoid the over-smoothing issue of the GCN model and to emphasize the original node features, we learned from the concept of ResNet [41] and added a layer of residual data to the GCN. To ensure the consistency between the dimension of the input feature and the node feature dimension of a layer GCN, we passed the initial input feature through an independent linear layer and directly connected it to the output of the GCN layer. Mathematically, the skip connection operation can be written as follows.

(3) $$H^{\left(p\right)}=H^{\left(1\right)}+\mathrm{relu}\;\left(\mathrm{linear}\left(X\right)\right)$$

where H^(1)^ represents the node features learned by the first GCN layer, and linear(X) denotes passing the initial input feature matrix into a linear layer.

The ERGCN model consists of two GCN layers. The activation function of the first GCN layer is Relu. We took the gene expression profiles of the samples as initial attributes and input them into the first GCN layer. Before feeding the output of the first GCN layer into the second GCN layer, we added the previous output with the initial node attributes that underwent a linear change to overcome over-smoothing (see Equation (3)). The second GCN layer adopted the softmax activation function, whose outputs are the probabilities of a patient belonging to every cancer subtype. We used the cross-entropy loss functions to quantify the cancer subtype prediction loss.


(4) $$\text{L}=-\overset{}{\underset{\text{d}\in y_{\text{T}}}{\sum }}\overset{F}{\underset{f=1}{\sum }}Y_{\text{df}}{\text{lnZ}}_{\text{df}}$$

Given a patient d in the training set T, Y_d__f_ is a sign function (0 or 1), Y_d__f_ equals one if the real class label of the patient d is one, otherwise, Y_d__f_ equals zero, Z_df_ is the predicted probability that cancer patient d is in the category f, which is the output of the second GCN layer. Support existed for a number F of cancer subtypes. The outputting dimension of the second GCN layer was F. We minimized the cancer subtype prediction loss to optimize the parameters in the ERGCN model.

In summary, Algorithm 1 illustrates the pseudocode of ERGCN.
**Algorithm 1 ERGCN** **Input:** Gene expression matrix $X\in R^{m\times n}$ with *m* number of samples whose vector length is *n*, corresponding true labels $Y\in R^{m}$, number of epochs e, learning rate η, dropout rate d. **Output:** Predicted labels Y. 1.Use Equation (1) to calculate the correlation between samples based on gene expression data to get the correlation matrix A (*m* * *m*). 2.Given a threshold θ, set the value of the matrix A greater than θ to 1, and set other values to 0 to obtain the adjacency matrix $\bar{A}$ (*m* * *m*). 3.For i = 1 to epochs do:
  H^(1)^ =ReLU( GCN1(X, $\bar{A}$)  H^(p)^ = H^(1)^ + ReLU(linear(X))  H^(2)^ = GCN2(H^(p)^, $\bar{A}$)  out = Softmax(H^(2)^)  Calculate the Loss by Equation (4).  Update the weights of ERGCN by gradient descent and back propagation.  end for 4.H^(1)^ =ReLU( GCN1(X, $\bar{A}$) 5.H^(p)^ = H^(1)^ + ReLU(linear(X)) 6.H^(2)^ = GCN2(H^(p)^, $\bar{A}$) 7.out = Softmax(H^(2)^) 8.Labels = out.max(dim = 1) 9.Return labels

### 2.4. Experimental Parameters

For ERGCN, we set the dimension of the first layer of the graph convolution to 64 and the dimension of the second layer of the graph convolution to the number of categories. We use the Adam optimizer function. The learning rate is set to 0.001. We performed 5-fold cross-validation ten times and compared the average results. The network structure of SAE was the input layer—500-200-50, the pre-training epochs of each layer were 20, and the final fine-tuning epochs were 40. The network structure of VAE was the input layer—512-512-128, with epochs of 300.

### 2.5. Assessing the Performance

#### 2.5.1. External Evaluation Metrics

The external evaluation index evaluates the effectiveness of the algorithm by comparing the predicted classification results with the real ones. We use the adjusted Rand index (ARI) to evaluate the distribution match between the classification results and the known benchmark subtypes. In addition to this, we also used the Accuracy, Precision, Recall, F1 Score, MCC indicators for comparison. The formulas of these indicators are as follows:

(5) $$\mathrm{Accuracy}\;=\frac{\mathrm{TP}+\mathrm{TN}}{\mathrm{TP}+\mathrm{TN}+\mathrm{FP}+\mathrm{FN}}$$

(6) $$\mathrm{Precision}=\frac{\mathrm{TP}}{\mathrm{TP}+\mathrm{FP}}$$

(7) $$\mathrm{Recall}=\frac{\mathrm{TP}}{\mathrm{TP}+\mathrm{FN}}$$

(8) $$F1\;\mathrm{Score}=\frac{2\mathrm{PR}}{P+R}=\frac{2\mathrm{TP}}{2\mathrm{TP}+\mathrm{FP}+\mathrm{FN}}$$

(9) $$\mathrm{MCC}=\frac{{\mathrm{TP}}^{*}\mathrm{TN}-{\mathrm{FP}}^{*}\mathrm{FN}}{\sqrt{\left(\mathrm{TP}+\mathrm{FP}\right)\left(\mathrm{TP}+\mathrm{FN}\right)\left(\mathrm{TN}+\mathrm{FP}\right)\left(\mathrm{TN}+\mathrm{FN}\right)}}$$

(10) $$\;\mathrm{ARI}=\frac{\mathrm{RI}-E\left(\mathrm{RI}\right)}{\max\left(\mathrm{RI}\right)-E\left(\mathrm{RI}\right)},\mathrm{RI}=\frac{\mathrm{TP}+\mathrm{TN}}{\mathrm{TP}+\mathrm{TN}+\mathrm{FP}+\mathrm{FN}},E\left(\mathrm{RI}\right)=\frac{\left(\mathrm{TP}+\mathrm{TN}\right)\left(\mathrm{TP}+\mathrm{FP}\right)}{\mathrm{TP}+\mathrm{TN}+\mathrm{FP}+\mathrm{FN}},\max\left(\mathrm{RI}\right)=\frac{\left(\mathrm{TP}+\mathrm{TN}\right)+\left(\mathrm{TP}+\mathrm{FP}\right)}{2}$$

where TP refers to positive samples predicted by the model as positive, TN refers to negative samples predicted by the model as negative, FP refers to negative samples predicted by the model as positive, and FN refers to positive samples predicted by the model as negative.

#### 2.5.2. Internal Evaluation Metrics

We used two internal evaluation metrics, Silhouette width and the Davies–Boulding Index(DBI), to assess the clustering quality without knowing the classification labels. The Silhouette width was calculated using Formula (11):

(11) $$\mathrm{Silhouette}\;\mathrm{width}\;=\frac{b\left(i\right)-a\left(i\right)}{\max\left(a\left(i\right),b\left(i\right)\right)}$$

where a(i) is the average distance between sample i and other samples of the same type, and b(i) is the average distance between sample i and all samples of different types. The value of the Silhouette width is in (−1,1). The larger the Silhouette width value, the higher the similarity of nodes within the same class, and the lower similarity of nodes between classes. The DaviesπBoulding Index formula was defined by Formula (12):

(12) $$\text{DBI}=\frac{1}{N}\sum_{i=1}^{N}\;\underset{i\neq \text{j}}{\text{max}}\left(\frac{\text{avg}\left(C_{i}\right)+\text{avg}\left(C_{j}\right)}{\text{dis}\left(C_{i},C_{j}\right)}\right)$$

where N is the number of clusters, avg(C_i_) is the average distance between sample i and its cluster centroid, dis(C_i_, C_j_) represents the distance between the center of class C_i_ and the center of class C_j_. The lower limit of the DBI is 0, and the smaller the DBI value, the better the clustering.

## 3. Results

To verify the effectiveness of ERGCN, we compared its cancer subtype prediction performance with some state-of-the-art methods in terms of internal and external evaluation indicators. The existing methods include stack autoencoder (SAE) [29], variational autoencoder (VAE) [30], Deeptype [35], GCN+PPI, support vector machine (SVM), Random Forest and GcForest [42]. VAE and SAE reduce the dimension of cancer gene expression data and learn the feature representation of cancer patients. Then, cancer patients are classified according to their learned feature representation through the classifiers SVM and GcForest [42]. Deeptype is the latest end-to-end method that combines supervised classification and unsupervised clustering to learn cluster structure representations for cancer-related data and identify cancer subtypes. GCN+PPI conducts a graph convolution operation on the PPI network to learn sample features and predict cancer subtypes [43]. We also investigated the effect of the correlation coefficient threshold on our model and experimented with new sample discrimination to verify the stability of our model. Finally, we performed difference analysis and functional enrichment analysis of cancer subtype-related genes on BRCA to explore potential cancer treatment targets.

### 3.1. Determination of Correlation Coefficient Threshold

**Figure 2:**
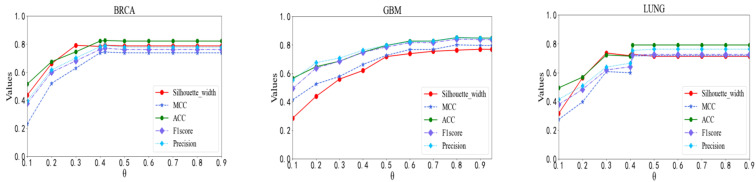
Performance comparison with respect to different correlation coefficient thresholds.

The first step of our model was to construct a patient network by calculating the PCC between patients. The parameter θ controls whether or not there is an edge connecting two patients. To test the effect of parameter θ, we compared the predictive performance of our model by setting θ to various values ranging from 0.1 to 0.9. We observed from Figure 2 that the performance of our model rose quickly with the increase of θ at the beginning. After that, it was relatively steady with different θ values. Our model performed best on the BRCA data set when θ was 0.42, where the ACC, MCC, F1 Score, Precision and Silhouette width reached 0.825, 0.743, 0.771, 0.790, and 0.792, respectively. Our model worked best on the GBM data set when θ was 0.8. Its ACC, MCC, F1 Score, Precision and Silhouette width achieved 0.851, 0.801, 0.841, 0.851, 0.763, respectively. On the LUNG data set, the performance of our model was relatively good when θ was 0.41. The ACC, MCC, F1 Score, Precision and Silhouette width were 0.792, 0.716, 0.722, 0.754, and 0.726, respectively. Hence, the parameter θ of our model was set to 0.42 in the BRCA data set, 0.8 in the GBM data set and 0.41 in the LUNG data set when we compared it with other methods.

### 3.2. Results of External Evaluation Metrics

We construct a sample similarity network using all samples. Then we divided all samples into five parts. Four parts were selected as the training set, and the remaining part was the test set. We took the average result of ten iterations of the 5-fold cross-validation test set as the evaluation metric. Table 1, Table 2 and Table 3 show the performance comparison between our model and other models regarding Accuracy, Recall, F1 Score, ACC, MCC, and ARI on the BRCA, GBM, and LUNG data sets. We observed the significant outperformance of our model compared with other models. On the BRCA data set, our Accuracy rate, F1 Score and MCC values were 2.94%, 14.48% and 5.03% higher than that of Gcforest, which has the best performance among the existing methods. Similar results were observed on the GBM and LUNG data sets. Our model’s Accuracy, F1 Score and MCC values ere 1.47%, 1.88% and 5.05% higher on the GBM dataset and 2.35%, 8.13%, 1.88% higher on the LUNG dataset compared to Gcforest, which works best among the existing methods on the two datasets.

### 3.3. Results of Internal Evaluation Metrics

**Figure 3:**
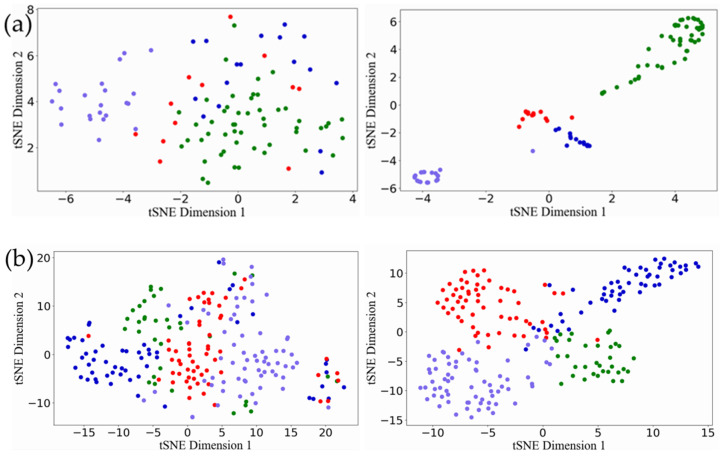

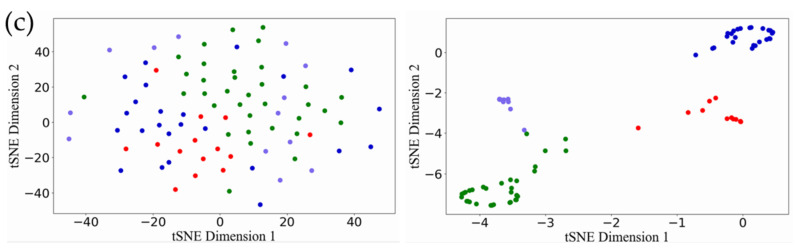
Visualization results of t-SNE. (**a**) The visualization result of BRCA. The first picture is the result of the original feature, and the second picture is the result of the latent features learned by ERGCN. (**b**) The visualization result of the GBM. The first picture is the result of the original feature. The second is the result of latent features learned by ERGCN. (**c**) The visualization result of LUNG, the first picture is the result of the original feature, and the second is the result of the latent feature learned by ERGCN.

We repeated the five-fold cross verification ten times. Table 4 reports the average Silhouette width and DBI of our model and other models for the test samples on the three data sets. The smaller the DBI index, the larger the Silhouette width and the more reasonable the classification result. It can be seen from Table 4 that our model keeps excellent performance in terms of compactness and separation. The Silhouette width of ERGCN reached 0.795, 0.763 and 0.727 on the BRCA, GBM and LUNG data sets, respectively, which was 17.24%, 34.32% and 24.49% higher than DeepType, which has the best internal evaluation values among the existing methods. Moreover, ERGCN resulted in 10.24%, 40.24% and 24.65% lower DBI values than DeepType on the BRCA, GBM and LUNG data sets, respectively. We use the t-SNE tool to visualize the initial features of the cancer samples in the test set and on their latent features learned by the ERGCN model. Figure 3 illustrates that the cancer samples can be separated into several subgroups well when using the features learned by our ERGCN model. These results prove the effectiveness of our model on cancer subtype classification.

### 3.4. Experiments with New Samples

ERGCN classifies cancer subtypes based on the sample similarity network, which calculates Pearson correlations between all pairs of samples ahead of time. To probe the performance of ERGCN on a new sample, which is a popular application in a clinical context of determining a person’s cancer subtype, we selected a single sample as the test set and regarded the remaining samples as the training set. We trained the model on the network built by the training samples. After that, we augmented the network by calculating the correlation between a new sample and other samples when testing the model. We collected the results of all single samples. Other methods only need to select a single sample as a test set and input the rest of the samples into the model as a training set. Table 5, Table 6 and Table 7 reports the average external evaluation indicators of our method and other methods across all individual samples. We noticed that ERGCN had good performance compared to other methods, with Accuracy values reaching 0.804, 0.850 and 0.824 on the BRCA, GBM and LUNG data sets, respectively.

**Table 5:** The experiment results for a new sample on BRCA dataset.

Methods	Precision	Recall	F1 Score	Accuracy	ARI	MCC
SAE+SVM	0.75693	0.54044	0.52594	0.72549	0.44104	0.57753
SAE+Gcforest	0.62501	0.55515	0.53656	0.73529	0.48863	0.59049
VAE+SVM	0.70438	0.68683	0.69412	0.75490	0.49052	0.62384
VAE+Gcforest	0.66973	0.65040	0.65643	0.76471	0.57447	0.63682
SVM	0.63776	0.51471	0.46261	0.72549	0.44240	0.59148
Gcforest	**0.84364**	0.64338	0.64064	0.79411	0.56589	0.68464
Random Forest	0.82441	0.62868	0.62397	0.78431	0.56030	0.66815
GCN+PPI	0.76280	0.68873	0.70813	0.79808	0.57397	0.69049
**ERGCN**	0.74755	**0.73884**	**0.73962**	**0.80392**	**0.62150**	**0.71075**

**Table 6:** The experiment results for a new sample on GBM dataset.

Methods	Precision	Recall	F1 Score	Accuracy	ARI	MCC
SAE+SVM	0.81642	0.81625	0.81538	0.81221	0.55023	0.74629
SAE+Gcforest	0.82595	0.82933	0.82708	0.82629	0.58206	0.76532
VAE+SVM	0.78944	0.78534	0.78682	0.79343	0.52562	0.72020
VAE+Gcforest	0.76416	0.75092	0.75601	0.76526	0.47159	0.68126
SVM	0.83590	0.82663	0.82985	0.83098	0.59225	0.77148
Gcforest	**0.85550**	0.80886	0.82051	0.83568	0.61514	0.77803
Random Forest	0.83445	0.80664	0.81572	0.82629	0.59218	0.76426
GCN+PPI	0.81691	0.81481	0.81547	0.82160	0.57898	0.75841
**ERGCN**	0.84325	**0.84021**	**0.84090**	**0.84977**	**0.64856**	**0.79738**

**Table 7:** The experiment results for a new sample on LUNG dataset.

Methods	Precision	Recall	F1 Score	Accuracy	ARI	MCC
SAE+SVM	0.53594	0.53283	0.49398	0.68235	0.41481	0.55207
SAE+Gcforest	0.65871	0.53268	0.52984	0.65882	0.34749	0.50912
VAE+SVM	0.78690	0.74056	0.75152	0.81176	0.63649	0.73533
VAE+Gcforest	0.63186	0.61147	0.60847	0.71764	0.52313	0.59664
SVM	0.58994	0.55804	0.52567	0.70588	0.46454	0.59018
Gcforest	**0.84865**	0.65167	0.63457	0.77647	0.58815	0.69397
Random Forest	0.58994	0.68130	0.63430	0.76235	0.56768	0.68308
GCN+PPI	0.61656	0.54185	0.55348	0.61176	0.23225	0.43827
**ERGCN**	0.79367	**0.78810**	**0.78903**	**0.82353**	**0.64861**	**0.75297**

### 3.5. Survival Analysis

**Figure 4:**
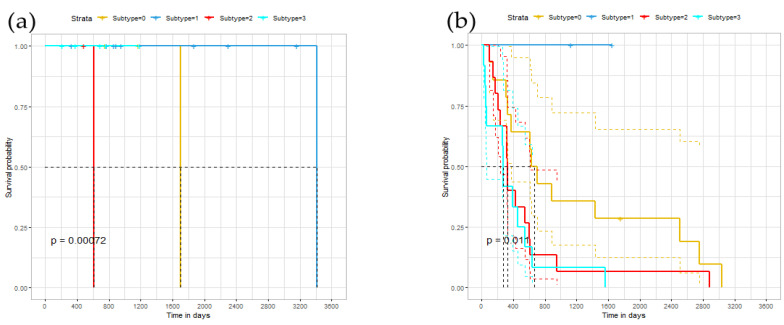

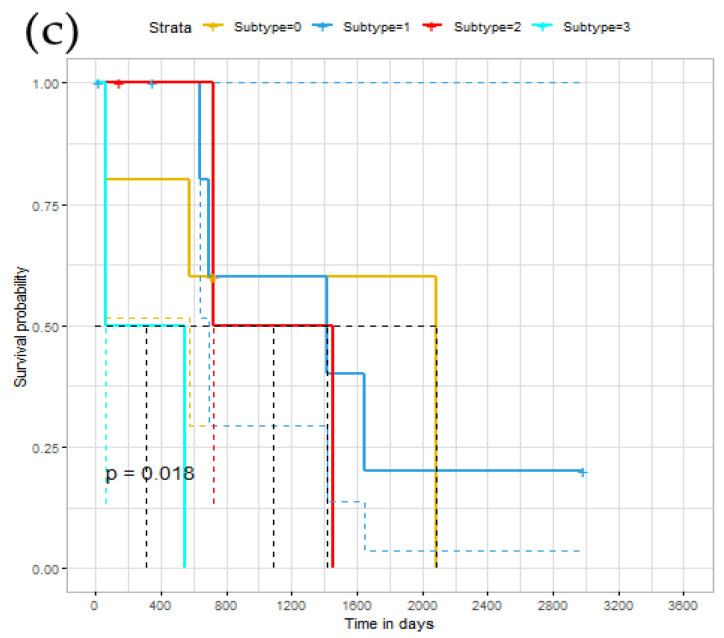
Survival time under different subtypes: (**a**) the survival curve of the BRCA data set; (**b**) the survival curve of the GBM data set; (**c**) the survival curve of the LUNG data set.

To further explore the relationship of identified subtypes, we conducted a survival analysis on the ERGCN results. Theoretically, different cancer subtypes should exhibit different survival curves. Figure 4 illustrates our model’s Kaplan–Meier survival curves on the BRCA, GBM and LUNG datasets, whose abscissa represents time, and the ordinate represents the observed survival rate. We also plotted the median survival time on the curve and calculated the *p*-value of the log-rank test on the survival curves of different subtypes. On the BRCA data set, the median survival time of the first group was 1699; the second group was 3418 days; the third group was 611 days; the fourth group was NA days. NA means that most patients in the fourth group cannot survive the median survival time. The distance between each category was very long. For GBM, the difference between the subgroups was not very obvious. The median survival time of the first group was 668 days; the second group was NA days; the third group was 327 days. The fourth group was 271 days. On the LUNG data set, the median survival time of cluster 1 was 2082 days; for cluster 2 it was 1415 days; for cluster 3 it was 1088 days, and for cluster 4 it was 306 days. The distance between each category was long. Hence, there was a significant difference in the survival curves of the cancer subtypes identified by our model on the three cancer datasets.

### 3.6. Ablation Study

ERGCN combines the GCN model and residual architecture to classify cancer samples. To investigate which parts contributed to ERGCN model’s excellent performance, we conducted an ablation study on the BRCA, GBM and LUNG datasets. MLP and GCN are two variations of our model. MLP reduces the dimensionality of cancer gene expression data through a two-layer neural network and then directly makes a classification through the softmax activation function. GCN leverages the same framework as ERGCN without using the residual architecture. The parameter settings of MLP and GCN were the same as those of ERGCN. Table 8 lists the comparison of their external evaluation metrics on the three datasets. On the BRCA dataset, compared with MPL and GCN, ERGCN yielded a 2.48% and 1.67% improvement to Accuracy values, 4.54% and 3.38% improvement to F1 Score values, and a 3.64% and 2.53% improvement to MCC values. Similar results were observed on the GBM and LUNG data sets. ERGCN also produced higher Accuracy, F1 Score and MCC values than its variations, MPL and GCN, on the two data sets, except a 0.4% lower F1 Score than MPL on the LUNG dataset. The observed improvement in the performance of ERGCN suggests that ERGCN successfully improves cancer subtype classification by conducting a residual graph convolutional operation on the sample similarity network.

### 3.7. Analyzing Key Genes of Breast Cancer Subtypes

**Figure 5:**
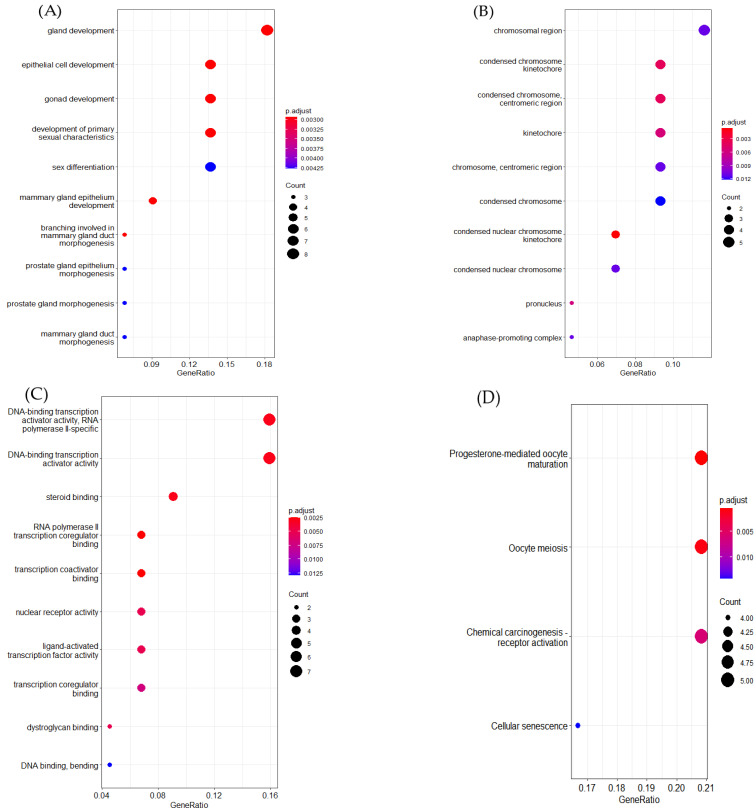
GO enrichment and KEGG enrichment analysis results. (**A**) Biological processes. (**B**) Cellular components. (**C**) Molecular functions. (**D**) KEGG enrichment.

In this part, we further probed the key genes that may cause the occurrence of breast cancer subtypes. According to Yang’s [16] approach, we leveraged ERGCN and the Random Forest model to determine the key genes of breast cancer subtypes. First, we ran the ERGCN model to predict a cancer subtype label for every sample in the BRCA dataset. Then we regarded the gene expression profile as the features of a sample and fitted a Radom Forest model with the sample features and their labels predicted by ERGCN. The Gini index measured the feature importance. We obtained 50 key genes with the highest Gini index scores which are essential in breast cancer subtype classification. To further investigate the function of these 50 key genes, we compared them with differentially expressed genes and found 37 key genes were differentially expressed. The differentially expressed genes were obtained using the R function TCGAanalyze-DEA, which compares tumor samples and normal solid tissue samples with the parameters dr.cut = 0.01 and logFC.cut = 1. Next, we employed the R package clusterProfiler [44] to perform GO and KEGG enrichment analysis for these 50 key genes (see Figure 5). GO enrichment studies the selected genes from three aspects: biological process (BP), cell composition (CC) and molecular function (MF).

In breast cancer, as for biological process, most of the selected genes were enriched in the regulation of gland development, epithelial cell development, gonad development, mammary gland epithelium development and primary sexual characteristics. For cellular components, the selected genes were mainly concentrated on the chromosomal region, cytoplasmic region and kinetochorer. For molecular function, the chosen genes were mostly located in DNA-binding transcription activator activity, RNA polymerase ll-specific, steroid binding and transcription coregulatori binding. The KEGG pathway analysis illustrated that most of the selected genes were enriched in the progesterone-mediated oocyte maturatiorl, oocyte meiosis, chemical carcinogenesis receptor activator and cellular senescence.

## 4. Discussion

This study designed a novel deep learning model, ERGCN, to classify cancer subtypes. Our contributions mainly lie in two aspects. One is that we considered the interactions between samples to make predictions. The other is that we introduced residuals to the end-to-end GCN model to avoid over-smoothing and to strengthen the original samples’ features. The observed improvement in the performance of ERGCN compared with other existing methods without using sample interactions suggests that interactions between samples contain rich and valuable information for cancer sample classification. Moreover, ERGCN performs best with a fair number of sample interactions (see the determination of correlation coefficient threshold). Our model also performed better than the GCN+PPI model by considering gene associations for cancer subtype classification. ERGCN outperformed two variations of our model, MLP and GCN, which proves that conducting a residual graph convolutional operation on the sample similarity network contributes to the prediction task. We also noticed that the models based on the autoencoder plus classifiers showed relatively lower performance. The two-stage method may not effectively learn features for cancer subtype identification compared with the end-to-end methods. In practice, we want to know which subtype a cancer sample belongs to, and to probe the subtype-related genes. We can combine ERGCN and the Random Forest model to determine the essential genes of a cancer subtype.

## 5. Conclusions

This work developed a cancer subtype identification method based on the residual graph convolutional network model. Firstly, we regarded gene expression profiles as sample features and constructed a sample similarity network according to their gene co-expression pattern. Next, we put the network and sample features into a residual graph convolutional network model to obtain the cancer subtype classification. Our method was applied to the three data sets of BRCA, GBM, and LUNG. The results show that our method was significantly better than other existing methods in terms of the internal and external evaluation indicators. We can see the stability of our model through the results of the new sample experiment. The survival of subtypes detected by our model differs considerably. The ablation study showed that the ERGCN combining the GCN model and residual architecture leads to higher performance than all its variants. Moreover, by analyzing the prediction results of breast cancer, we find some key genes that may cause the occurrence of breast cancer subtypes, demonstrating the practicality of ERGCN.

However, our model still has some limitations. On the one hand, our model relies on a predefined threshold to construct the sample similarity network. Too high or too low threshold values may affect the performance of our model. However, it is hard to set proper threshold automatically. On the other hand, we only used gene expression profiles to classify samples. Other omics data may provide complementary information for cancer subtype classification [45]. Hence, our future work will design a more practical approach to stratify cancer subtypes by integrating multi-omics data.

## Figures and Tables

**Table 1:** The external evaluation metrics of every model on BRCA dataset.

Methods	Precision	Recall	F1 Score	Accuracy	ARI	MCC
SAE+SVM	0.46688	0.47171	0.41178	0.568	0.23869	0.36318
SAE+Gcforest	0.47257	0.47837	0.43852	0.64067	0.30144	0.43627
Deeptype	0.60228	0.62621	0.59160	0.753	0.57466	0.64430
VAE+SVM	0.66438	0.65065	0.63436	0.74114	0.48724	0.60771
VAE+Gcforest	0.64519	0.64151	0.61159	0.77448	0.54275	0.65777
SVM	0.42288	0.51925	0.45485	0.72076	0.43940	0.57969
Gcforest	0.64539	0.65442	0.62575	0.79638	0.57676	0.69289
Random Forest	0.66267	0.66451	0.63839	0.78952	0.57012	0.68508
GCN+PPI	0.64554	0.62277	0.61171	0.75005	0.49454	0.62303
**ERGCN**	**0.78953**	**0.79844**	**0.77055**	**0.82576**	**0.62873**	**0.74322**

**Table 2:** The external evaluation metrics of every model on GBM dataset.

Methods	Precision	Recall	F1 Score	Accuracy	ARI	MCC
SAE+SVM	0.79338	0.79440	0.78250	0.79355	0.52781	0.72323
SAE+Gcforest	0.79924	0.78409	0.77825	0.78831	0.51550	0.71606
Deeptype	0.78081	0.75975	0.74804	0.77300	0.50885	0.79063
VAE+SVM	0.80097	0.78549	0.78055	0.79761	0.52952	0.72786
VAE+Gcforest	0.77588	0.76140	0.75264	0.77575	0.51302	0.70079
SVM	0.83716	0.81796	0.81292	0.82083	0.56993	0.76267
Gcforest	**0.85667**	0.82310	0.82187	0.83661	0.61136	0.78249
Random Forest	0.85179	0.81486	0.81643	0.83511	0.61546	0.78059
GCN+PPI	0.81717	0.79781	0.79759	0.80755	0.55226	0.74441
**ERGCN**	0.85109	**0.84795**	**0.84065**	**0.85131**	**0.64321**	**0.80066**

**Table 3:** The external evaluation metrics of every model on LUNG dataset.

Methods	Precision	Recall	F1 Score	Accuracy	ARI	MCC
SAE+SVM	0.62703	0.64589	0.60029	0.70706	0.40495	0.59873
SAE+Gcforest	0.50461	0.53870	0.48131	0.63412	0.30443	0.49150
Deeptype	0.65217	0.66711	0.62727	0.736	0.53235	0.64140
VAE+SVM	0.71101	0.68801	0.67435	0.75177	0.48261	0.65223
VAE+Gcforest	0.70152	0.67114	0.64492	0.74588	0.49020	0.65056
SVM	0.46486	0.53482	0.46342	0.67176	0.44398	0.55509
Gcforest	0.68092	0.68020	0.64116	0.76823	**0.58791**	0.69718
Random Forest	0.66950	0.68130	0.63430	0.76235	0.56768	0.68308
GCN+PPI	0.59129	0.568	0.55040	0.65412	0.30853	0.51357
**ERGCN**	**0.75400**	**0.74699**	**0.72242**	**0.79176**	**0.57377**	**0.71602**

**Table 4:** The internal evaluation metrics of every model.

Methods	BRCA		GBM		LUNG	
	DBI	Silhouette Width	DBI	Silhouette Width	DBI	Silhouette Width
SAE+SVM	2.0001	−0.0056	2.5358	0.0402	1.9491	−0.0005
SAE+Gcforest	1.8179	0.0335	2.4135	0.0465	2.0028	0.0222
DeepType	0.39641	0.62221	0.75048	0.42000	0.57735	0.48204
VAE+SVM	2.1105	−0.0132	2.9650	−0.0376	1.8451	−0.0270
VAE+Gcforest	1.9178	0.0444	2.8630	−0.0455	1.7715	−0.0147
SVM	2.15145	0.11750	2.77210	−0.00830	2.66726	0.00047
Gcforest	1.96480	0.06851	2.80126	0.00025	2.30813	−0.00803
Random Forest	1.98764	0.05645	2.81110	-0.00069	2.28595	−0.00269
GCN+PPI	2.02747	0.03644	2.91481	0.00961	2.25382	−0.0148
**ERGCN**	**0.29402**	**0.79463**	**0.34806**	**0.76318**	**0.33086**	**0.72691**

**Table 8:** The experiment results for the ablation study.

	BRCA			GBM			LUNG		
	MLP	GCN	ERGCN	MLP	GCN	ERGCN	MLP	GCN	ERGCN
Precision	0.74126	0.76061	**0.78953**	0.84129	0.84435	**0.85109**	**0.76058**	0.74748	0.754
Recall	0.75557	0.7641	**0.79844**	0.84113	0.84397	**0.84795**	**0.74867**	0.73711	0.74699
F1 Score	0.72517	0.73677	**0.77055**	0.83316	0.83556	**0.84065**	**0.72696**	0.71772	0.72242
Accuracy	0.80095	0.80904	**0.82576**	0.84285	0.84525	**0.85131**	0.78941	0.78941	**0.79176**
ARI	0.60001	0.60768	**0.62873**	0.62204	0.6292	**0.64321**	0.55106	0.56351	**0.57377**
MCC	0.70687	0.71806	**0.74322**	0.78966	0.79278	**0.80066**	0.70979	0.70839	**0.71602**

## Data Availability

The datasets supporting the conclusions of this article are available at https://github.com/weiba/ERGCN/tree/master.
